# A Novel Pale-Yellow Coat Color of Rabbits Generated *via* *MC1R* Mutation With CRISPR/Cas9 System

**DOI:** 10.3389/fgene.2019.00875

**Published:** 2019-09-18

**Authors:** Ning Xiao, Hongli Li, Laiba Shafique, Shanshan Zhao, Xiaoping Su, Yu Zhang, Kuiqing Cui, Qingyou Liu, Deshun Shi

**Affiliations:** State Key Laboratory for Conservation and Utilization of Subtropical Agro-bioresources, Guangxi University, Nanning, China

**Keywords:** rabbit, *MC1R*, novel coat color, Cas9, knockout

## Abstract

Coat color is of great importance in animal breed characteristics; it is not only a significant productive trait but also an indispensable economic trait, especially in the rabbit industry. In the present study, the relationship between melanocortin 1 receptor (*MC1R*) genotypes and coat color phenotypes was observed in five rabbit breeds with popular coat colors that are present in China. These breeds comprised the Lianshan black rabbit (BR), Fujian yellow rabbit (YR), New Zealand white rabbit (WR), Gray Giant rabbit (GR), and Checkered Giant rabbit (CR), which were firstly determined, and the results showed that GR had an E allele; WR, CR, and BR had a 6-bp in-frame deletion (c.281_286del6, E^D^ allele); and YR had a 30-bp deletion (c.304_333del30 E allele). To explore the feasibility of obtaining a novel rabbit coat color through the mutation of *MC1R* with the CRISPR/Cas9 system, two single-guide RNAs (sgRNAs) were designed for the *MC1R* gene, and the editing efficiency was confirmed by injection of rabbits’ zygotes. Unlike the donor rabbits whose coat color was originally black, two novel pale-yellow-coated rabbits were generated in the founders. A total of six novel *MC1R* gene deletions were identified in the two founder rabbits, in which the longest deletion was more than 700 bp. The histological hematoxylin-and-eosin (H&E) staining results indicated that eumelanin amounts were absent in hair follicles of *MC1R*-knockout (KO) rabbits, when compared with that of donor BR. In addition, the messenger RNA (mRNA) levels of some key downstream genes in the MC1R pathway were all downregulated in *MC1R*-KO rabbits compared with BR and YR. These results further indicate that loss-of-function MC1R contributed to blocking the synthesis of eumelanin and created a novel pale-yellow coat color in the *MC1R*-KO rabbits, and gene editing technology may be a useful tool to generate novel phenotypes in rabbit breeding.

## Introduction

Animal coat color is one of the most important breed characteristics, which is not only a significant productive trait but also an indispensable economic trait. As an important genetic marker for molecular breeding, great attention has been paid to the inheritance of rabbit coat color for a long time. In mammals, the *Extension* locus encodes the melanocortin 1 receptor (MC1R), which is mainly expressed in hair follicle and skin melanocyte, closely related to skin pigmentation. The activation of α-melanocyte-stimulating hormone (MSH) initiates with MC1R complex signaling that leads to the manufacturing of black and dark-brown eumelanin pigments. The agouti signaling protein (ASIP) antagonized the receptors in dissimilar pathways, causing a switch from a eumelanin type pigment to phaeomelanin (Lu et al., 1994; Ollmann et al., 1998) and producing yellow or red pigments. *MC1R* is a highly polymorphic gene. Mutations in some single-nucleotide polymorphism (SNP) loci have been shown in the alteration of mammalian coat color, such as red guinea pig (Cone et al., 1996), chestnut horse (Marklund et al., 1996), and Holstein cow with red skin (Joerg et al., 1996). Additionally, *MC1R* gene mutations linked with different coat colors have been described in humans (Valverde et al., 1995), mice (Robbins et al., 1993), pigs (Kijas et al., 1998; Kijas et al., 2001), cattle (Klungland et al., 1995; Rouzaud et al., 2000), sheep (Vage et al., 1999), goats (Fontanesi et al., 2009), dogs (Everts et al., 2000; Newton et al., 2000), chickens (Kerje et al., 2003), and foxes (Vage et al., 1997; Vage et al., 2005), in which functional mutations increased to produce black/dark coat color and lack of functions usually generated original/yellow coat color.

In domesticated rabbits (*Oryctolagus cuniculus*), conventional genetic studies have been involved in crossbreeding experiments of breeds of different coat colors where five alleles were recognized at the *Extension* locus, representing the following: E^D^ shows black dominant; E^S^ represents steel, which is weaker version of E^D^; E wild type (WT) expresses normal gray or an extension of black; e^J^ exists in Japanese brindling with a mosaic distribution of yellow and black; and e expresses a red/yellow appearance with white belly. The E^D^ > E^S^ > E > e^J^ > e order showed the dominance (Robinson, 1958; Fox, 1994). Fontanesi et al. (2006) identified in-frame deletion, connected with two possibilities, which were recessive red (c.304_333del30; allele e) and black dominant (c.280_285del6; alleles E^D^ and/or E^S^) coat colors *via* sequencing nearly the complete coding sequence (CDS) of the rabbit *MC1R* gene. Awareness about the *MC1R* gene’s role and mechanism in coat color pattern determination predominantly relied on analysis of spontaneous mutations in farm animals. Genome-specific manipulation is important to further explore the *MC1R* gene function and to provide insight into the coat color mechanism with alteration and formation by precise modification in the *MC1R* gene.

Though it is widely known that gene polymorphism of *MC1R* is related to the coat color of animals, most studies only focused on the correlation between spontaneous mutations and the phenotype in various animal species. There is still lack of effective *MC1R*-knockout (KO) animal models to further study the function of *MC1R*. Here, by using the CRISPR/Cas9 genome editing system, we can destroy the protein structure of *MC1R* partially or completely, which may help us to better understand the mechanism of this gene. Moreover, this is the first research using this system to create a mammalian model and study the function of MC1R, and the use of the CRISPR/Cas9 system makes it possible to artificially obtain animals with popular or new coat colors.

In an attempt to find out whether the phenotype produced by the artificially modified *MC1R* gene is consistent with that of the spontaneous loss-of-function mutation, we knocked out the *MC1R* gene of a Lianshan black rabbit (BR) through a dual-sgRNA-directed CRISPR/Cas9 system and generated two *MC1R* mutated rabbits with pale yellow coats, which were distinctly different from the wild yellow rabbits. In our present work, it is the first time to find out a novel coat color in rabbits by the interruption of the *MC1R* gene *via* CRISPR/Cas9. *MC1R*-KO rabbit can be used as a model to further study *MC1R* gene information on different coat colors.

## Materials and Methods

### Rabbit Sampling

Ear clips of five individual rabbits [one BR, one YR, one New Zealand white rabbit (WR), one Gray Giant rabbit (GR), and one Checkered Giant rabbit (CR)] were sampled, and the genomic DNA was extracted from tissue lysate by using phenol–chloroform and recovered by alcohol precipitation.

### Cloning and Analysis of Rabbit *MC1R*

For cloning and analysis of rabbit *MC1R* gene, genomic DNA from BR, YR, WR, GR, and CR with equal amounts (100 ng) was used as template for PCR amplification by a pair of specific primers (*MC1R*-F:5′-GGTGGCTGGTGTGGAAATGT-3′ and *MC1R*-R:5′-GCTGGCAAAGGGGCACTA-3′), which were designed based on the sequence of rabbit *MC1R* (GenBank: FN658676.1). A gel extraction kit (Omega Bio-Tec, USA) was used for PCR product purification and for cloning into the pMD18-T vector (Takara, Japan). Ten positive clones of each rabbit (a total of 50 clones) were sequenced and then analyzed by using BioEdit and MEGA7. Besides, the CDS of the *MC1R* gene of two other breeds, Thrianta rabbit (GenBank: FN658678.1) and Japanese brindling (GenBank: FN658679.1), was added in multiple-sequence alignment.

### SgRNA Design and Plasmid Construction

In our previous published data, the protocols for sgRNA design and vector construction have been discussed in detail (Su et al., 2018). A dual-sgRNA targeting rabbit *MC1R* gene was designed according to the multiple-sequence alignment result of rabbits in different colors, and these two target sites avoided the spontaneous deletion regions in the rabbit *MC1R* gene.

### *In Vitro* Transcription of Cas9 mRNA and sgRNA

The hSpCas9 (CMV-T7-NLS-hSpCas9-NLS) expression plasmid was linearized by restriction endonuclease *Eco*RI preparing for hSpCas9 messenger RNA (mRNA) transcription *in vitro*. sgRNAs with a T7 promoter sequence (TAATACGACTCACTATAGG) in upstream were made to produce sgRNA by *in vitro* transcription. The templates used to generate the sgRNA were obtained by using the primers listed in **Table S1**. The linearized plasmid of hSpCas9 and the amplified T7-sgRNA product were subjected to gel purification and applied as the template for *in vitro* transcription, respectively, by a mMESSAGEmMACHINE^®^ T7 Kit (Ambion, USA) and a MEGAshortscript™ T7 Kit (Ambion, USA) in strict accordance with each manufacturer-recommended protocol. The quality and the concentration of RNA were estimated by agarose gel electrophoresis and determined by a BioSpec-nano UV-Vis spectrophotometer (Japan), respectively. The Cas9 mRNA and the sgRNA were both purified through a MEGAclear^TM^ transcription clean-up kit (Ambion, USA), eluted into RNase-free water, and frozen at −80 °C.

### Embryo Collection, Microinjection, and Transplantation

Superovulation was conducted in 6–8-month-old female BR with 1.2-mg follicle-stimulating hormone (FSH) at intervals of 12 h for six times, after mating with male BR and then injected with 100-IU human chorionic gonadotropin (hCG). Eighteen hours after hCG injection, rabbit embryos were collected at the pronucleus stage by flushing the oviducts with a 5-ml oocyte manipulation medium. A mixture of *in vitro* transcribed Cas9 mRNA (100 ng/μl) and sgRNAs (20 ng/μl per sgRNA) was microinjected into the cytoplasm of fertilized eggs. The injected zygotes were transferred into an embryo culture medium and cultured at 38.5 °C in 5% CO_2_ for 5–10 min. About 14–16 injected embryos were then autologously transplanted into the oviduct of the donor rabbit.

### Mutation Efficiency Detection in Blastocysts and Pups by PCR and Sequencing

For verification of indel mutations, injected embryos were collected at the blastocyst stage. Genomic DNA was extracted from a single blastocyst with a cell lysis buffer (Ambion) at 75 °C for 15 min and 95 °C for 10 min. The sgRNA target sites were amplified by using high-fidelity Golden Star T6 DNA polymerase (TsingKe, Beijing, China). PCR primers used for mutation detection were as follows: F, 5′-ACAGCCTCCCCCAGTCCT-3′; R, 5′-GCACCTCCTTGAGCGTCC-3′. The genomic DNA from *MC1R* KO and WT newborn rabbits were extracted from a small sample of ear tissue using Tris-phenol/chloroform. Primers *MC1R*-F/R were used for PCR genotyping. After gel purification, the PCR products were cloned into a pEASY-blunt simple vector (TransGen, Beijing, China) and then subjected to Sanger sequencing. Mutations were identified *via* alignment of the sequenced alleles to the WT.

### Prediction of the Modified MC1R Protein Structure of *MC1R* KO Rabbits

To assess the influence of targeted deletion, five major targeted modifications were selected. Their 3D models were constituted using the online program Phyre2 (http://www.sbg.bio.ic.ac.uk/phyre2/html/page.cgi?id=index). The two spontaneous alleles of the MC1R protein were set as the control.

### Off-Target Analysis

By testing whether off-target mutations presented in *MC1R*-KO rabbits, we predicted the potential off-target sites (POTS) of the sgRNAs using the online CRISPR Design Tool developed by the Zhang group at Massachusetts’s Institute of Technology (http://crispr.mit.edu/), and we selected the top eight POTS for each sgRNA. Then the PCR products of these POTS were subjected to T7E1 assay and Sanger sequence analysis. All primers for off-target assay were listed in **Table S2**.

### T7E1 Cleavage Assay

The PCR products of POTS were gel purified. Next, a solution comprising 5-μl PCR products, 1.1–μl NEBuffer 2.1, and 4.4-μl ultrapure water was incubated at 95 °C for 10 min and then annealed at room temperature for at least 30 min. After that, hybridized PCR products were digested with 5-IU T7E1 (NEB) for 30 min at 37 °C and finally subjected to 2.5% agarose gel electrophoresis.

### Histology Analysis

Skin tissues of BR and *MC1R*-KO rabbits were fixed with 4% paraformaldehyde for 24 h at 4 °C. The increasing concentrations of dehydrated ethanol (70% for 7 h, 85% for 5 h, 90% for 2 h, 95% for 2 h, and 100% for 2 h), dealcoholized with xylene and embedded in paraffin, were used for histological examination. The paraffin-embedded tissues which were sectioned into 5-μm-thick slices were stained with hematoxylin and eosin (H&E) and analyzed with a fluorescence inverted microscope (Nikon, Japan). The integral optical density (IOD) analysis of histological sections was performed by Image-Pro Plus 6.0 software.

### Real-Time Quantitative PCR (qRT-PCR)

Total RNA was extracted from the skins of BR, YR, and *MC1R*-KO rabbits by TRIzol reagent (Ambion, Life Technologies, USA) according to the manufacturer’s protocol, then treated with DNase I (Thermo Scientific, USA), and reverse-transcribed into complementary DNA (cDNA) using the RevertAid First Strand cDNA Synthesis Kit (Thermo Scientific). qPCR was performed using ChamQ™ Universal SYBR® qPCR Master Mix (Vazyme Biotech, Nanjing, China) with the ABI PRISM 7500 Real Time System (Applied Biosystems, USA), and the relative gene expression normalized to the glyceraldehyde 3-phosphate dehydrogenase (GAPDH) was determined by the 2^−ΔΔCT^ formula. All the data of gene expression were performed three times. The candidate genes were microphthalmia-associated transcription factor (*MITF*), tyrosinase (*TYR*), tyrosinase-related protein 1 (*TYRP1*), and dopachrome tautomerase (*DCT*). The specific primers for qPCR were listed in **Table S3**.

### Statistics

All the quantitative data are expressed as mean ± SEM, with at least three individual determinations in all experiments. The data were analyzed by *t*-test using the SPSS 17.0 program. A probability of *p* < 0.05 was considered statistically significant.

## Results

### The Relation of *MC1R* Genotypes and Coat Color Phenotypes

**Figure 1:**
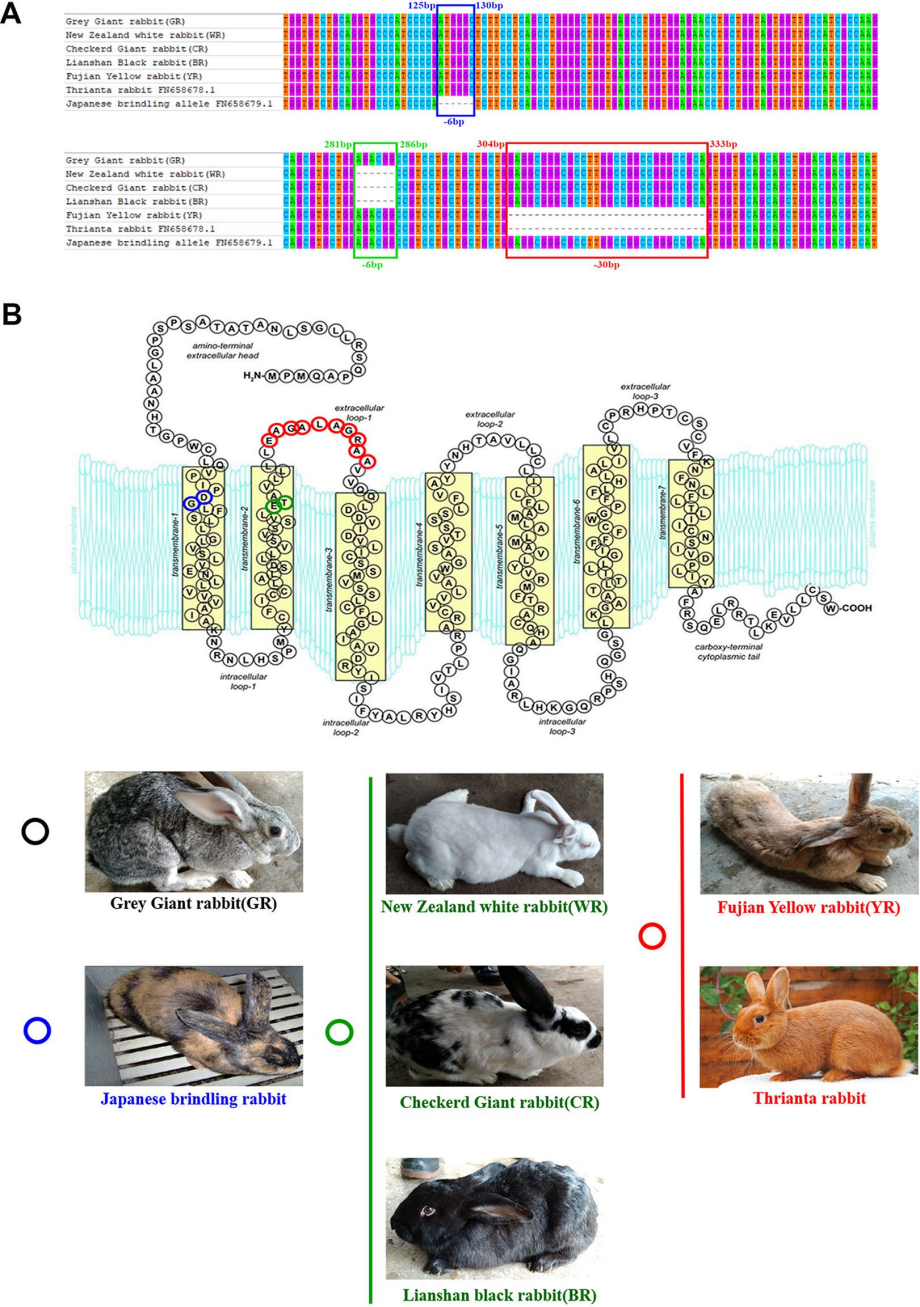
Multiple-sequence alignment of rabbit *MC1R* gene and protein structures. **(A)** Multiple-exon-sequence alignment of *MC1R* gene of Gray Giant rabbit (GR), New Zealand White rabbit (WR), Checkered Giant rabbit (CR), Lianshan black rabbit (BR), Fujian yellow rabbit (YR), Thrianta rabbit (GenBank: FN658678.1), and Japanese brindling (GenBank: FN658679.1).The six nucleotides in blue and green boxes are those that are deleted in the rabbit *MC1R* gene c.125-130del6 and c.281-286del6 alleles, respectively. The 30 nucleotides in the red box are those deleted in the c.304-333del30 alleles. **(B)** Spontaneous mutations carried by rabbits of different breeds and coat colors. The *MC1R* gene of GR are wild-type alleles which encoded 317 amino acids. The two amino acids in blue circle is those deleted in the c.125-130del6 allele (e^J^), carried by Japanese brindling rabbit. The two amino acids in green circle are those deleted in the c.125-130del6 alleles (E^D^), carried by WR, CR, and BR. And the 10 amino acids in red circle are those that are deleted in the c.304-333del30 alleles (e), carried by YR and Thrianta rabbit (the diagram of amino acid composition of rabbit mature MC1R protein with seven transmembrane domains was modified from Wolf Horrell et al. (2016).

Cloned separately were 1,409 bp (regarded as a WT sequence) of the *MC1R* gene (the whole CDS of 954 bp was amplified and sequenced, 362 bp of the 5′-untranslated region and 93 bp of the 3′-untranslated region) in five rabbits with different coat colors, namely, BR, YR, WR, GR, and CR. The sequences obtained from WR, CR, and BR identified the presence of the 6-bp in-frame deletion (c.281_286del6, allele E^D^). The c.304_333del30 was identified in YR and Thrianta rabbit as the determinant of the e allele. Another 6-bp deletion flanked by a G > A transition in 5′ (c.[124G > A;125_130del6]) existed in Japanese brindling allele e^j^ (**Figure 1A**). The *MC1R* gene of GR was WT allele E, which encoded 317 amino acids (AA). The E^D^ allele carried by WR, CR, and BR had a two-amino-acid deletion in the second transmembrane (TM) domain. The e allele carried by YR and Thrianta rabbit had a 10-amino-acid deletion in the first extracellular loop. And the Japanese brindling allele (e^j^) had two AA in the first TM domain (**Figure 1B**). These three mutations were spontaneous.

### Design and Determine the Efficiency of sgRNA Targeting Rabbit *MC1R* Gene

**Figure 2:**
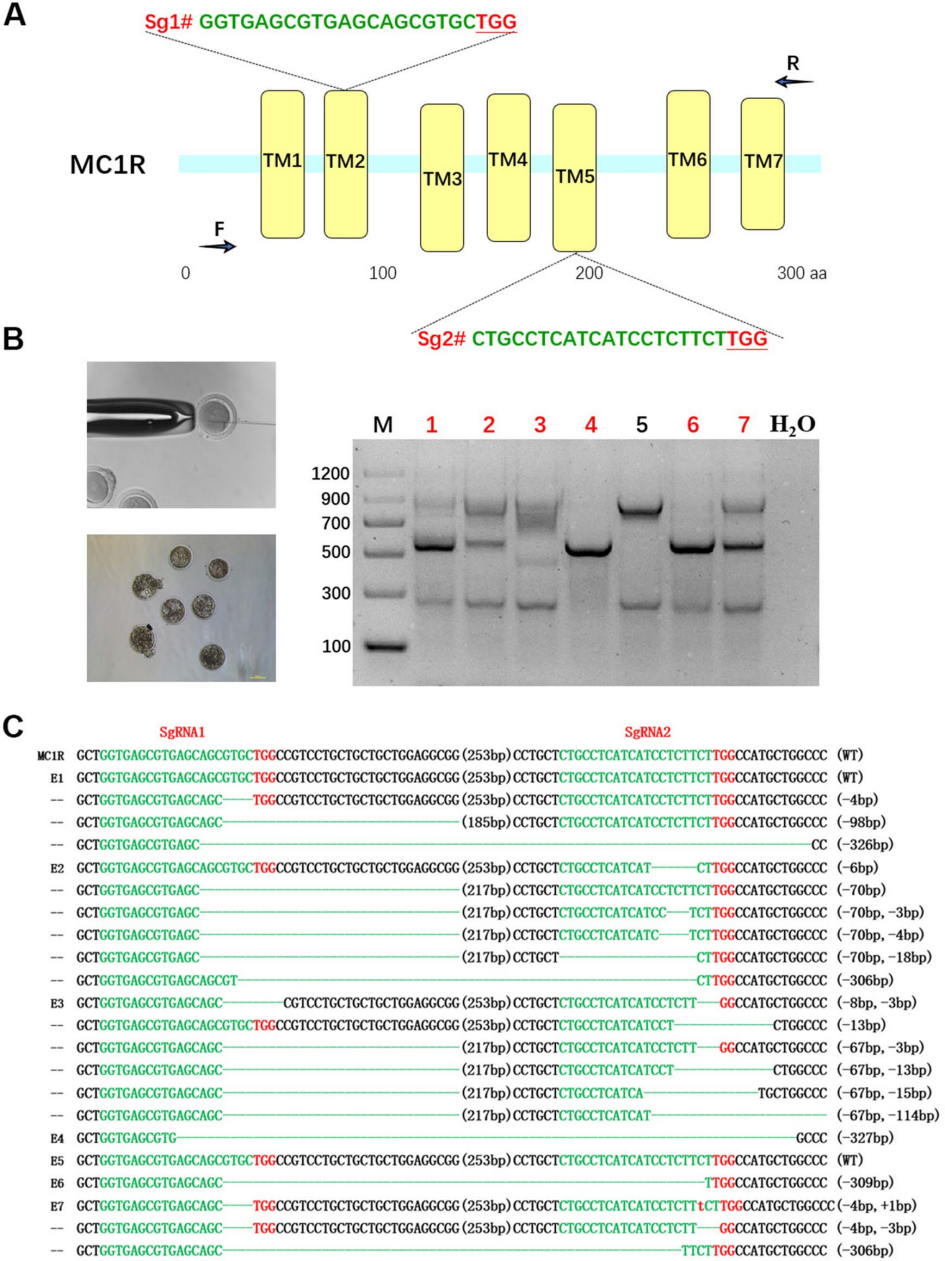
Dual single-guide RNA (sgRNA)-directed deletion of *MC1R* in zygotes. **(A)** Schematic diagram of sgRNA targeting the rabbit *MC1R* gene loci. The yellow rectangle represents the transmembrane domain of MC1R. Two sgRNA sequences, sgRNA1 (Sg1#) and sgRNA2 (Sg2#), are highlighted in green. Protospacer adjacent motif (PAM) sequences are presented in red with underline. Primers F and R are used for mutation detection in embryos. **(B)** Cytoplasmic injection of zygotes using the CRISPR/Cas9 system. Seven blastocysts are collected. Mutation detection in blastocyst by PCR. M, marker; numbers 1–7 represent different blastocysts used in this study. The number in red represents the positive embryos. Scale bar, 100 μm. **(C)** T-cloning and Sanger sequencing of the modified *MC1R* alleles in blastocysts. Wild-type sequence is shown at the top of the targeting sequence. Sequences of sgRNAs are marked in green, the PAM sequences are in red, insertions are highlighted in lowercase red letters, and deletions are designated by dashes. E: embryos; WT: E^D^ allele; deletion: “−”; insertion: “+”.

For the purpose of destroying *MC1R* in BR, a dual sgRNA targeting the CDS of *MC1R* was designed according to the multiple-sequence alignment result of rabbits in different colors, which avoided the natural deletion regions in rabbit *MC1R* gene (**Figure 2A**). Since the existence of spontaneous mutations, we did not perform the T7E1 assay in this determination, and the editing results were confirmed by Sanger sequence analysis. As shown in **Figures 2B, C**, mutations were identified in six of these seven blastocysts (85.7%). There were different long-fragment deletions in every mutated blastocyst. These results indicated that a dual–sgRNA-directed CRISPR/Cas9 system efficiently knocked out rabbit *MC1R* in our study.

### Generation of *MC1R*-KO Rabbits Using CRISPR/Cas9 System

**Figure 3:**
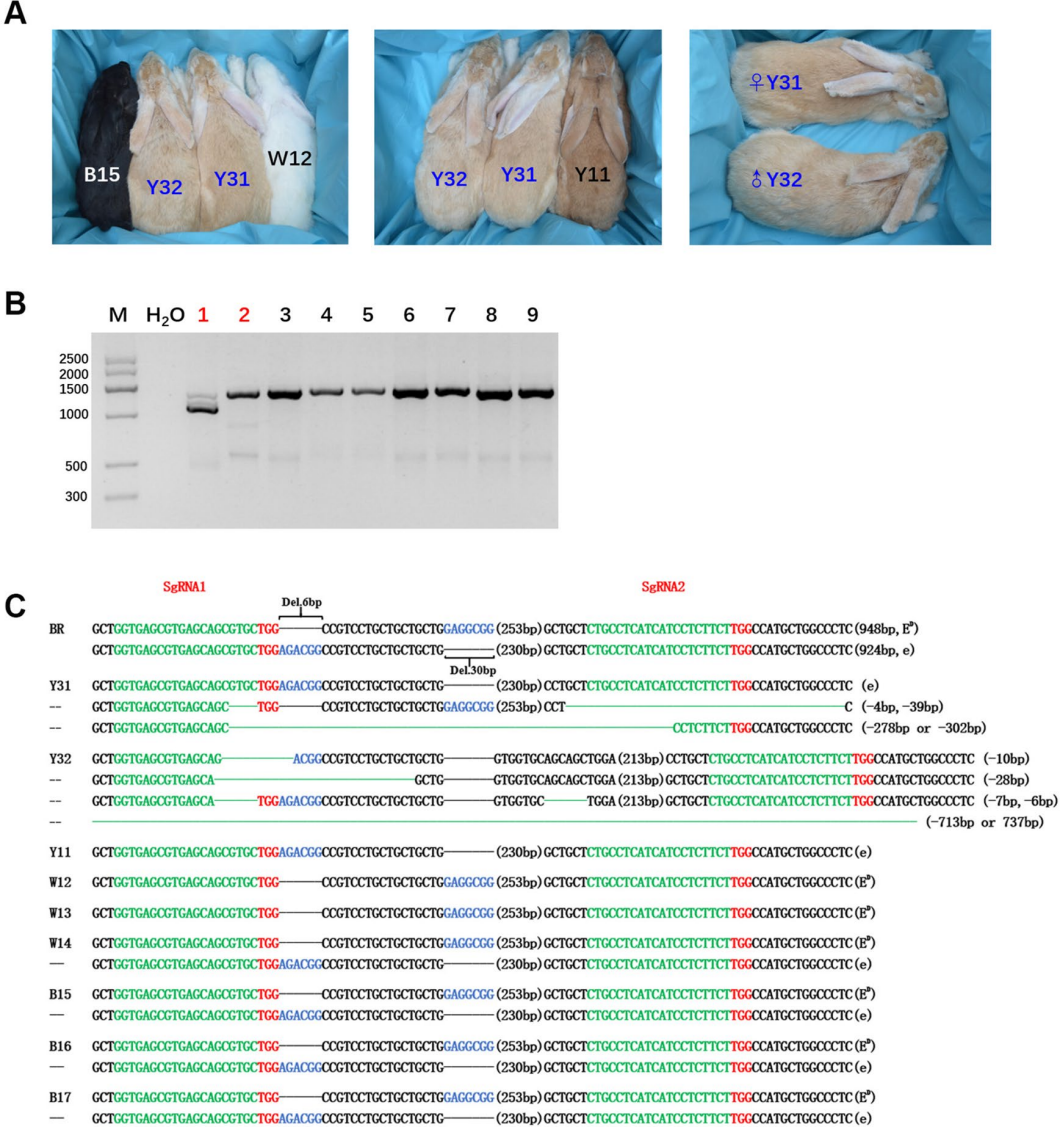
Generation of the *MC1R*-knockout (KO) rabbits with a new coat color using the CRISPR/Cas9 system. **(A)** Photographs of the *MC1R*-KO rabbits at 6 months old generated by the CRISPR/Cas9 system; rabbit B15, black; rabbit W12, white; rabbit Y11, yellow; and rabbit Y31 and Y32, pale yellow. Y31 is a female rabbit, and Y32 is male. **(B)** The mutation was determined in founder rabbits by PCR. M, marker; 1, Y31; 2, Y32; 3, Y11; 4, W12; 5, W13; 6, W14; 7, B15; 8, B16; and 9, B17. The number in red represents the *MC1R* mutated pups. **(C)** T-cloning and Sanger sequencing in nine pups with modified *MC1R* alleles. The two alleles E^D^ and e of parent Lianshan black rabbits (BR) are shown at the top of the targeting sequence. Spontaneous deletions are designated by black dashes, and the corresponding nondeleted bases are shown in blue. Sequences of single-guide RNAs (sgRNAs) are marked in green, the protospacer adjacent motif (PAM) sites are highlighted in red, insertions are highlighted in lowercase red letters, and deletions are designated by green dashes. Deletion: “−”.

After embryo transplantation and full-term gestation, two pregnant rabbits successfully gave birth to nine live pups (**Table 1**). *MC1R* modifications were not found in the seven pups from mother rabbit #1, but the other two pups from mother rabbit #3 all had a mutated *MC1R* gene. As expected, large-fragment deletions of *MC1R* gene were present in these two pups numbered Y31 and Y32, and Y31 had one WT sequence, namely, e allele, compared with BR, but Y32 did not (**Figures 3B, C**). As shown in **Figure 3A**, under the natural condition, the black male and female parents who had the heterozygous genotype (E^D^/e) would have pups of three colors, black (B15), yellow (Y11), and white (W12). Interestingly, the *MC1R*-KO rabbits Y31 and Y32 showed a novel beautiful coat color, a pale yellow distinctly different from wild yellow. And we found that two founder rabbits carried multiple mutant genotypes, ranging from 10-bp deletion to over 700-bp deletion. Because the deleted fragment is too long, which included both the 6-bp and 30-bp spontaneous deletions, it was impossible to know the original genotype and original coat color of Y32. But one thing was certain, that Y31 carried the E^D^ allele at first, which meant that it was originally black and this black coat color was changed to pale yellow *via* gene editing. Taken together, we successfully generated *MC1R*-KO rabbits with a novel coat color using CRISPR/Cas9.

**Table 1:** Summary of the *MC1R*-knockout (KO) rabbits generated by CRISPR/Cas9.

	sgRNA	gRNA/Cas9 mRNA (ng/μl)	Embryos injected	Embryos transferred	Pregnancy	Pups obtained (% transferred)	*MC1R*-KO pups (% pups)	Pups with color change
1#	Sg1 + Sg2	40/100	13	13	Yes	7 (53.8%)	0	0
2#	Sg1 + Sg2	40/100	17	16	No	–	–	–
3#	Sg1 + Sg2	40/100	14	14	Yes	2 (14.3%)	2 (100%)	2
4#	Sg1 + Sg2	40/100	14	14	No	–	–	–

### Characterization of Disruptive Modifications of *MC1R*-KO Rabbits

**Figure 4:**
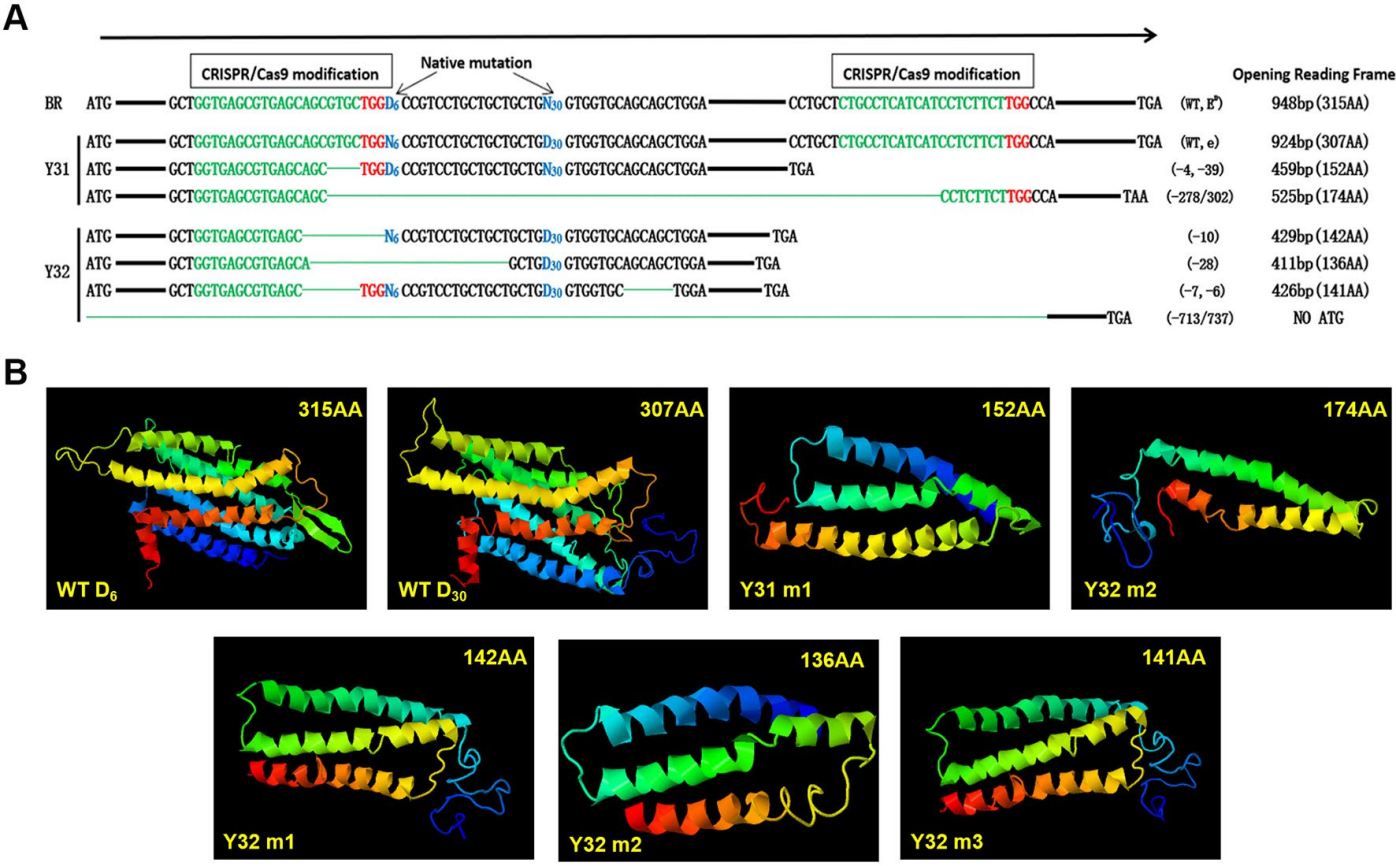
Diagram of the spontaneous deletions (D_6_N_30_ or N_6_D_30_) and prediction of protein structure of single-guide (sgRNA)/Cas9 targeted modifications of two *MC1R*-knockout (KO) rabbits. **(A)** “D_6_” is designated as a spontaneous deletion (versus N_6_ as nondeletion), with 6-bp deletion downstream of the ATG start codon. “D_30_” is designated as spontaneous deletion (versus N_30_ as nondeletion), with 30-bp deletion downstream of D_6_. The CRISPR/Cas9 targeted modifications are illustrated separately in the upstream and downstream of spontaneous deletion diagram, which shows the forms of modifications, and predicted protein products of a premature stop codon of amino acids. The sgRNA sequences are marked in green, PAM sites are red, and the deletions are designated by green dashes. **(B)** Construction of MC1R E^D^ models according to five modifications and two WT types of BR. WT D_6_, structure of MC1R E^D^ allele; WT D_30_, structure of MC1R e allele; Y31 m1, editing type of −4 and −39 bp; Y31 m2, editing type of −278 or 302 bp; Y32 m1, editing type of −10 bp; Y32 m2, editing type of −28 bp; Y32 m3, editing type of −7 and −6 bp.

Through sequencing analysis, there were two types of *MC1R* modifications found in Y31 and four types in Y32. As there is involvement of a spontaneous 6-bp deletion denoted as D_6_ (c.281_286del6) and a 30-bp deletion denoted as D_30_ (c.304_333del30) in *MC1R* CDS, for a more intuitive display, we genotyped all modifications of two targeted rabbits by D_6_ or D_30_ deletions compared with BR (D denotes deletion and N nondeletion). Previous investigations found that D_6_N_30_ spontaneous mutations, namely, E^D^ allele, were dominantly presented in black rabbit, and N_6_D_30_, namely, e allele, was recessive red/yellow (Fontanesi et al., 2006). So the genotype of the black one was E^D^/−, and the yellow one was ee. As shown in **Figure 4A**, the genotype of Y31 was E^D^/e, and that of Y32 was −/e, both of which are uncertain because the two spontaneous mutations were all deletion. Therefore, it was certain that the coat color of Y31 was changed from black to pale yellow *via* the CRISPR/Cas9 system. Further analysis revealed that, except the fourth modified type of Y32, which was deleted too long to have a start codon, the rest of the modifications all resulted in a frame shift and premature stop codon and, as consequence, led to a truncated MC1R protein that could completely or partially abolish the function of MC1R. The result of protein structure prediction showed that premature termination caused the absence of most of the TM structures which were essential for MC1R function (**Figure 4B**). It can be deduced that the targeting events led to the disruption of the MC1R function.

### Off-Target Detection in the *MC1R*-KO Rabbits

**Figure 5:**
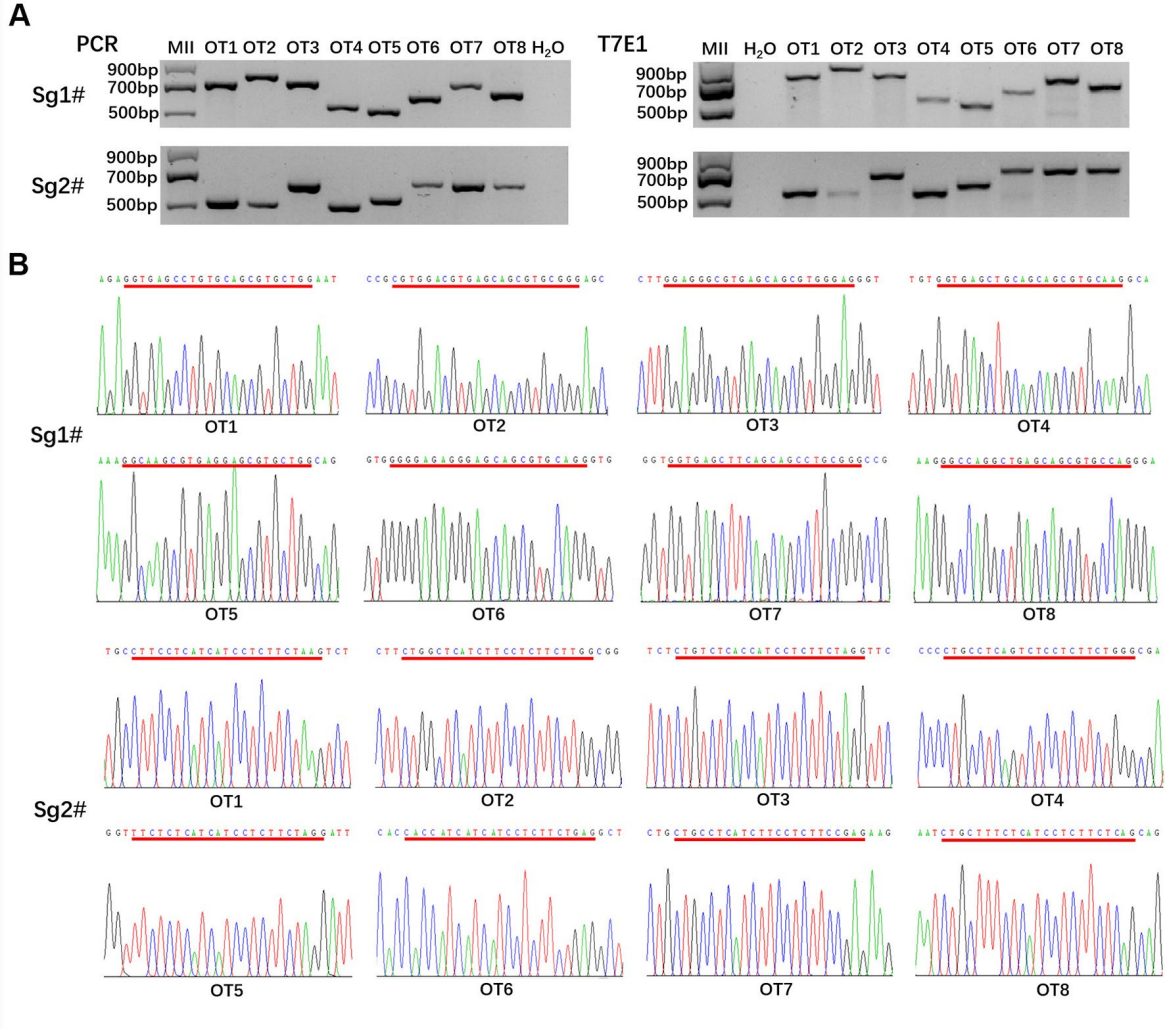
Off-target detection in the *MC1R*-knockout (KO) rabbits. **(A)** PCR and T7E1 assays of the top eight potential off-target sites (POTS) for Sg1# and Sg2#, respectively. No fragment is found in T7E1 assays. **(B)** Chromatogram sequence analysis of 16 POTS for Sg1# and Sg2# using PCR products in the founders. No double curve was shown in any sequencing diagrams. The POTS are underlined in red.

To detect whether off-target mutations were present in *MC1R*-KO rabbits, we predicted POTS of the two sgRNAs using the online CRISPR Design Tool developed and selected the top eight POTS for each sgRNA. The results of T7E1 assay and Sanger sequence analysis showed that none of the off-target mutations was detected at these POTS in the *MC1R*-KO rabbits (**Figures 5A, B**). The information about POTS was listed in **Table S2**.

### Phenotype Assessment of *MC1R*-KO Rabbits and Signal Pathway Analysis

**Figure 6:**
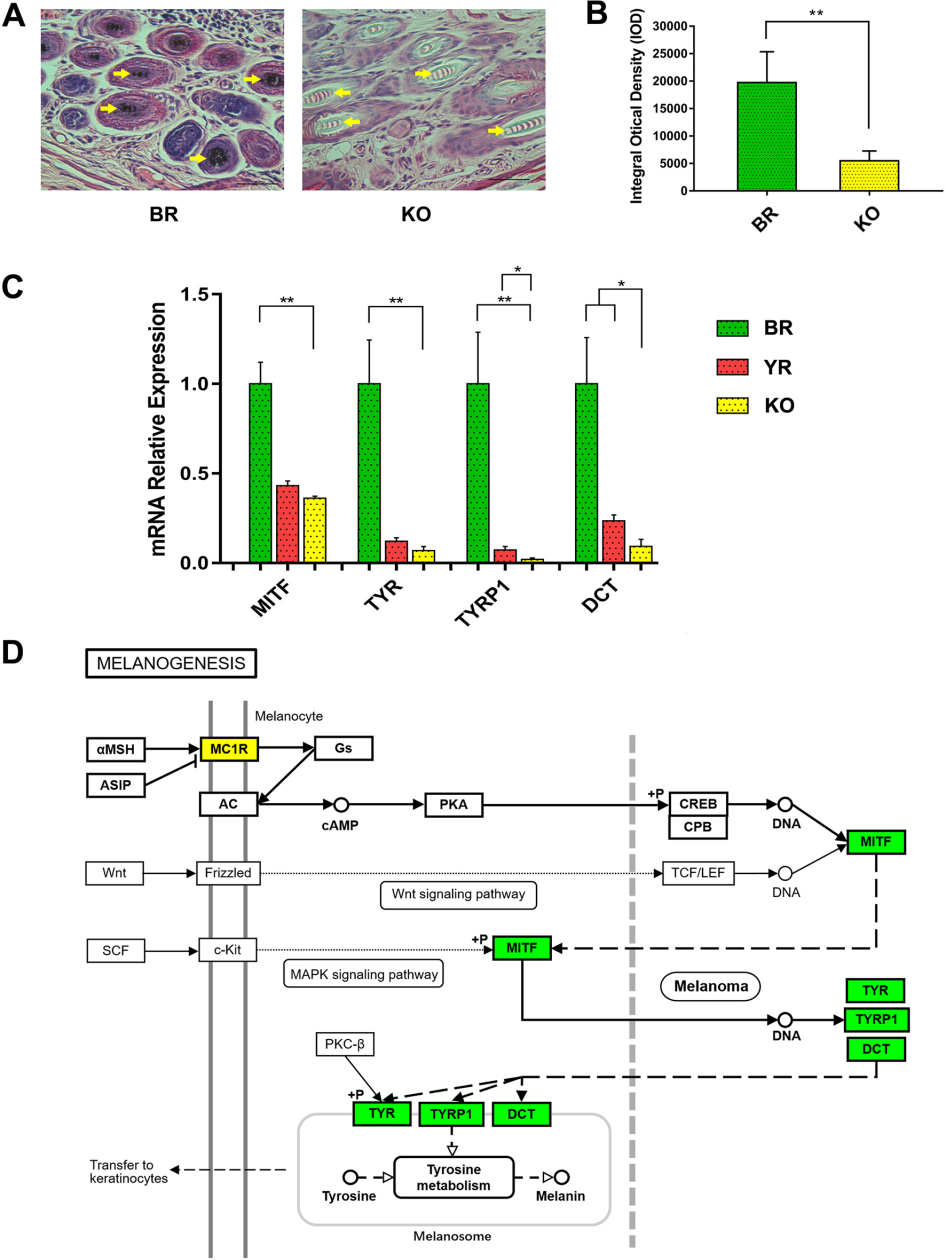
Phenotype identification and the expression of downstream genes in MC1R signal pathway of *MC1R*-KO rabbits. **(A)** H&E staining of the skin from the BR and KO rabbits. BR, Lianshan black rabbit; H&E, hematoxylin and eosin; KO, *MC1R*-knockout rabbits. Scale bar, 100 μm for 400×. **(B)** Integral optical density (IOD) analysis of histological sections by Image-Pro Plus software. **(C)** Relative expression of *MITF*, *TYR*, *TYRP1*, and *DCT* genes were determined by Real-time quantitative PCR (qRT-PCR). Bar graphs show mean values. Error bars represent standard error (SE). **(D)** The relation of *MITF*, *TYR*, *TYRP1*, and *DCT* in the melanogenesis pathway. The genes with a green frame are significantly downregulated in the KO skin compared with the BR skin. BR, Lianshan black rabbit; YR, Fujian yellow rabbit; KO, *MC1R*-KO rabbit. *0.01 < *p* < 0.05; **p < 0.01 (the diagram of melanogenesis pathway was simplified from the KEGG pathway database, 04916, 10/23/15, Kanchisa Laboratories).

The histological H&E staining results indicated that eumelanin amounts were not present in hair follicles of *MC1R*-KO rabbits, when compared with BR (**Figure 6A**). And the histogram showed that the IOD of histological sections of KO rabbits was significantly lower than that of BR (**Figure 6B**). We further examined whether disruption of the MC1R led to reduced expression of the downstream gene of the MC1R signal pathway (**Figure 6D**) in the skin tissue. As shown in **Figure 6C**, the mRNA levels of *MITF*, *TYR*, *TYRP1*, and *DCT* gene were significantly reduced in *MC1R*-KO rabbits, when compared with the control group of BR. These results further indicate that loss of MC1R protein function contributed to blocking of the synthesis of eumelanin and created a new coat color in the *MC1R*-KO rabbits.

## Discussion

MC1R is formed of seven α-helical (TM) domains with a DRY motif at the junction of the third TM domain, an intracellular C-terminus associated with palmitoylation site, and an extracellular N-terminus attached with an N-linked glycosylation site, like other G-protein-coupled receptors (GPCRs) (Yang, 2011). The extracellular N-terminal tail performed the following functions: (1) ligand affinity (Chhajlani et al., 1996) and (2) sign anchor (Wallin and von Heijne, 1995; Garcia-Borron et al., 2005). There is a conserved cysteine residue present at the N-terminus junction and the first TM domain which is entirely essential for receptor function (Frandberg et al., 2001). The C-terminus in GPCR often plays a role in protein trafficking from the endoplasmic reticulum to the plasma membrane (Schulein et al., 1998; Qanbar and Bouvier, 2003) and also in receptor interactions with the G protein at the plasma membrane (Strader et al., 1994). Previous studies have demonstrated that mutations which interrupt the pentapeptide or especially in the tripeptide variant such as premature termination at R306 (Newton et al., 2000) or removal of the terminal pentapeptide (Sanchez-Mas et al., 2005) result in minimized plasma membrane MC1R expression. as the extracellular loops of MC1R interconnect with ligands, mutations in this region affect binding affinity (Chhajlani et al., 1996). Likewise, mc1r intracellular loops play a vital role in binding of the Gs protein. Thus, any loss of function from *MC1R* gene mutations could affect the MC1R signaling pathway and interfere with melanogenesis.

The WT E allele of rabbit *MC1R* gene has 317 AA, and the phenotype of coat color is normal grey, like GR. The E^D^ and E^S^ alleles remove two AA in the second TM domain (c.280_285del6) and result in the dominant black and steel coat colors, respectively (Fontanesi et al., 2006). Therefore, the black breeds like BR certainly carry this dominant allele (E^D^/E^D^ or E^D^/−).The recessive allele removes 10 AA of the first extracellular loop (c.304_333del30) and was in a homozygous condition in all red/yellow rabbit breeds such as Thuringian breeds and YR (Fontanesi et al., 2006). In our study, the result of sequencing the complete *MC1R* gene CDS of GR, WR, CR, BR, and YR was consistent with previous reports. It is reasonable to hypothesize that the *MC1R*-KO gene of BR will produce a yellow phenotype.

In an attempt to find out whether the phenotype produced by the artificially modified *MC1R* gene is consistent with that of the spontaneous loss-of-function mutation, the *MC1R* gene was targeted by the CRISPR/Cas9 system. By coinjection of sgRNA and Cas9 mRNAs into rabbit zygotes, we got two *MC1R*-KO rabbits of novel coat color. Sanger sequencing indicated that the deletion in the *MC1R* gene of the founder rabbits ranged from 10 to more than 700 bp. The *MC1R*-KO model in mammals was the first time to be reported. Compared with the wild yellow rabbit, the coat color of *MC1R*-KO rabbits was obviously lighter (**Figure 3A**). It is worth noting that in offspring, white rabbits were produced besides black and yellow ones, and all white rabbits carried the dominant black allele E^D^ (**Figure 3C**). In rabbits, the white coat color is controlled by the C locus which encodes the *Tyrosinase* (*Tyr*) gene rather than the E locus that encodes *MC1R* (Aigner et al., 2000). Previous studies have demonstrated that *TYR* is the rate-limiting enzyme to catalyze melanogenesis and plays key roles in melanin biosynthesis and albinism (Simeonov et al., 2013). Mutated *Tyr* gene resulted in a lack of pigmentation because of melanin production deficiency. We speculated that the parent rabbits carried the heterozygous *Tyr* mutation and the albinism phenotype was not affected by whether *MC1R* was knocked out or not.

In order to analyze the original genotypes of *MC1R*-KO rabbits, we also identified the *MC1R* genotypes of parent rabbits. The genotypes of parents were all E^D^/e, and the progenies from mother rabbit #1 showed three expected genotypes. One of two *MC1R*-KO rabbits could not be determined due to the very-large-fragment deletions in the *MC1R* gene, but the other originally carrying the dominant black allele E^D^ was confirmed. Due to the nonhomologous end joining (NHEJ) feature caused by CRISPR/Cas9, alleles were randomly edited and may have different modifications for each allele. There were six types of modifications in *MC1R*-KO rabbits, which resulted in frame shift and premature termination, and even part of intron sequences had even been deleted. Based on the 3D model prediction of protein, premature terminations all consequently led to protein truncation, and all the five truncated proteins lacked at least half of the TM domains. As for two spontaneous mutant proteins, D_30_ lacked two β-turns compared with the protein of D_6_. β-Turns play a vital part in proteins, which makes it possible to change the direction for the polypeptide, concerning protein folding and molecular recognition (Rose et al., 1985). On account of the protein encoded by the e allele missing two β-turns in the first extracellular loop, the recognition of MC1R by ligands was affected; thus, the activation of the pathway to produce eumelanin was interfered with. It is reasonable to hypothesize that the destruction of TM domains and the complete C-terminal domain produced by the KO of the *MC1R* gene destroyed the receptor structure to a greater extent, which resulted in more functional defects of MC1R.

MC1R is involved in melanin synthesis by binding to two ligands: α-MSH and ASIP (Choudhary et al., 2016). The combination of α-MSH to MC1R will activate the adenylate cyclase system, increase the cyclic adenosine monophosphate (cAMP) level, and then promote the synthesis of eumelanin and inhibit the yield of pheomelanin, whereas binding of ASIP to MC1R reduces constitutive signaling to the cAMP pathway, which results in a decrease in the production of eumelanin and an increase in the yield of pheomelanin, thus regulating the change of coat color (Le Pape et al., 2008;Le Pape et al., 2009). As the downstream genes of MC1R/cAMP pathway, *MITF* (Steingrimsson et al., 2004; Levy et al., 2006), *TYR* (Wang and Hebert, 2006; Ando et al., 2007), *TYRP1* (del Marmol and Beermann, 1996; Lai et al., 2018), and *DCT* (Aroca et al., 1992; Guyonneau et al., 2004) genes were very crucial in the melanogenesis pathway (**Figure 6D**). We demonstrated that the MC1R/cAMP pathway was disturbed and dysfunctional MC1R leads to different degrees of reduction in the expression of these genes in *MC1R*-KO rabbits compared with the wild yellow rabbit (**Figure 6C**). This result reasonably explained the lighter yellow phenotype of *MC1R*-KO rabbits at the molecular level.

## Conclusion

In summary, a novel pale-yellow coat color in *MC1R*-KO rabbits was generated by interruption of the *MC1R* gene in BR *via* CRISPR/Cas9 system. Our work demonstrated that rabbit *MC1R* gene plays a vital role in coat color determination and disruption of *MC1R* gene will change the coat color. The gene editing rabbits with the novel coat color can be used as a model for further study on the mechanisms of *MC1R* gene function. In addition, our study established the foundation of using the CRISPR/Cas9 system to artificially obtain animals with popular or new coat colors.

## Data Availability

All datasets for this study are included in the manuscript and the **Supplementary Files**.

## Ethics Statement

Rabbits were used in present study were fed regularly at Laboratory Animal Center of Guangxi University. All experimental studies were approved and reviewed by the Experimental Animal Care and Use Committee of Guangxi University (Permit code: GXU2019-029). In present study, rabbit experiment was performed by the Principle Guidance for the Use and Care of Laboratory Animals.

## Author Contributions

NX, DS, and QL conceived and designed the experiments. NX, HL, SZ, XS, and YZ performed the experiments. NX analyzed the data, and LS wrote the manuscript. KC revised the manuscript. All authors read and approved the final version of the manuscript.

## Funding

The present study was granted and supported by the National Natural Science Foundation of China (Grant Nos. 31772597 and 31860638), and we are also grateful to the Guangxi Natural Science Foundation (Grant Nos. AA17204051 and AB16380042).

## Conflict of Interest Statement

The authors declare that the research was conducted in the absence of any commercial or financial relationships that could be construed as a potential conflict of interest.
