# Supplementary material for: A Novel Pale-Yellow Coat Color of Rabbits Generated via MC1R Mutation With CRISPR/Cas9 System
Source: Front Genet. 2019 Sep 18;10:875. doi: 10.3389/fgene.2019.00875 (PMC6759607; doi:10.3389/fgene.2019.00875)
Supplement: Supplementary file 1 [file Table_1.docx]

**Table S1. Primers used for obtaining templates of sgRNA with T7 promoter.**

| Name | Sequence (5’ to 3’) |
| --- | --- |
| T7-Sg1# | TAATACGACTCACTATAGGGGGTGAGCGTGAGCAGCGTGC |
| T7-Sg2# | TAATACGACTCACTATAGGGCTGCCTCATCATCCTCTTCT |
| T7-Down | AAAAAAAGCACCGACTCGGTGCCA |

**Table S2. 16 potential off-target sites examined by PCR and primers used for list.**

|  | **20** | **19** | **18** | **17** | **16** | **15** | **14** | **13** | **12** | **11** | **10** | **9** | **8** | **7** | **6** | **5** | **4** | **3** | **2** | **1** | **N** | **G** | **G** |
| --- | --- | --- | --- | --- | --- | --- | --- | --- | --- | --- | --- | --- | --- | --- | --- | --- | --- | --- | --- | --- | --- | --- | --- |
| **MC1R sg1#** | **G** | **G** | **T** | **G** | **A** | **G** | **C** | **G** | **T** | **G** | **A** | **G** | **C** | **A** | **G** | **C** | **G** | **T** | **G** | **C** | **T** | **G** | **G** |
| **OTS 1** | **G** | **G** | **T** | **G** | **A** | **G** | **C** | **C** | **T** | **G** | **T** | **G** | **C** | **A** | **G** | **C** | **G** | **T** | **G** | **C** | **T** | **G** | **G** |
| **OTS 2** | **C** | **G** | **T** | **G** | **G** | **A** | **C** | **G** | **T** | **G** | **A** | **G** | **C** | **A** | **G** | **C** | **G** | **T** | **G** | **C** | **G** | **G** | **G** |
| **OTS 3** | **G** | **G** | **A** | **G** | **G** | **G** | **C** | **G** | **T** | **G** | **A** | **G** | **C** | **A** | **G** | **C** | **G** | **T** | **G** | **G** | **G** | **A** | **G** |
| **OTS 4** | **G** | **G** | **T** | **G** | **A** | **G** | **C** | **T** | **G** | **C** | **A** | **G** | **C** | **A** | **G** | **C** | **G** | **T** | **G** | **C** | **A** | **A** | **G** |
| **OTS 5** | **G** | **G** | **C** | **A** | **A** | **G** | **C** | **G** | **T** | **G** | **A** | **G** | **G** | **A** | **G** | **C** | **G** | **T** | **G** | **C** | **T** | **G** | **G** |
| **OTS 6** | **G** | **G** | **G** | **G** | **A** | **G** | **A** | **G** | **G** | **G** | **A** | **G** | **C** | **A** | **G** | **C** | **G** | **T** | **G** | **C** | **A** | **G** | **G** |
| **OTS 7** | **G** | **G** | **T** | **G** | **A** | **G** | **C** | **T** | **T** | **C** | **A** | **G** | **C** | **A** | **G** | **C** | **C** | **T** | **G** | **C** | **G** | **G** | **G** |
| **OTS 8** | **G** | **G** | **C** | **C** | **A** | **G** | **G** | **C** | **T** | **G** | **A** | **G** | **C** | **A** | **G** | **C** | **G** | **T** | **G** | **C** | **C** | **A** | **G** |

|  | Site | Potential Off Target Site | Position | No. of Mismatches | Gene | PCR Primer (5’ to 3’) | Product Size(bp) |
| --- | --- | --- | --- | --- | --- | --- | --- |
| Sg1# | OT1 | GGTGAGCCTGTGCAGCGTGCTGG | Chr2:  +95042062 | 2 | - | F: TTTGCAACTCACTGCCCTCT  R: CCTTTGTGTCTTTGCCTGGTG | 707 |
|  | OT2 | CGTGGACGTGAGCAGCGTGCGGG | Chr12:  +33652570 | 3 | TMEM  151B | F: ACTGCTACTTCCGCCTCTTTC  R: CGGGACACCAGAGTAAGGAG | 772 |
|  | OT3 | GGAGGGCGTGAGCAGCGTGGGAG | chrUN0:  +53260732 | 3 | STK10 | F: GACAGAGGGAGGTGAAAGGC  R: GGGTCCTAGCTGTCATGTCG | 715 |
|  | OT4 | GGTGAGCTGCAGCAGCGTGCAAG | chrUN0:  -951264 | 3 | - | F: CCTTTCGGTTCTCTGTGCTCT  R: CTGTCCATGGCGCTTGTATG | 531 |
|  | OT5 | GGCAAGCGTGAGGAGCGTGCTGG | chr1:  +103377309 | 3 | - | F: GCACCCTTTCCCCGTAACT  R: GTGGTCCCTGTTGTGGGTTC | 511 |
|  | OT6 | GGGGAGAGGGAGCAGCGTGCAGG | chr14:  +39467157 | 3 | - | F: TCGCTTTAGTTTAGGTGGAAGTGT  R:CAGCACTGCAGGTTAGAGGTTTAG | 607 |
|  | OT7 | GGTGAGCTTCAGCAGCCTGCGGG | chrUN0:  +143328002 | 3 | GPR153 | F: TCTTCCTTCCCACAGGCAGT  R: TCTTGCTCTTGCTGGACCTTC | 738 |
|  | OT8 | GGCCAGGCTGAGCAGCGTGCCAG | chr17:  -14779856 | 4 | - | F: TTGAGAAACTGCCAAGCTGA  R: GCACAATACTCACCCCTAACC | 636 |

|  | **20** | **19** | **18** | **17** | **16** | **15** | **14** | **13** | **12** | **11** | **10** | **9** | **8** | **7** | **6** | **5** | **4** | **3** | **2** | **1** | **N** | **G** | **G** |
| --- | --- | --- | --- | --- | --- | --- | --- | --- | --- | --- | --- | --- | --- | --- | --- | --- | --- | --- | --- | --- | --- | --- | --- |
| **MC1R sg2#** | **C** | **T** | **G** | **C** | **C** | **T** | **C** | **A** | **T** | **C** | **A** | **T** | **C** | **C** | **T** | **C** | **T** | **T** | **C** | **T** | **T** | **G** | **G** |
| **OTS 1** | **C** | **T** | **T** | **C** | **C** | **T** | **C** | **A** | **T** | **C** | **A** | **T** | **C** | **C** | **T** | **C** | **T** | **T** | **C** | **T** | **A** | **A** | **G** |
| **OTS 2** | **C** | **T** | **G** | **G** | **C** | **T** | **C** | **A** | **T** | **C** | **T** | **T** | **C** | **C** | **T** | **C** | **T** | **T** | **C** | **T** | **T** | **G** | **G** |
| **OTS 3** | **C** | **T** | **G** | **T** | **C** | **T** | **C** | **A** | **C** | **C** | **A** | **T** | **C** | **C** | **T** | **C** | **T** | **T** | **C** | **T** | **A** | **G** | **G** |
| **OTS 4** | **C** | **T** | **G** | **C** | **C** | **T** | **C** | **A** | **T** | **T** | **C** | **T** | **C** | **C** | **T** | **C** | **T** | **T** | **C** | **T** | **G** | **G** | **G** |
| **OTS 5** | **T** | **T** | **C** | **T** | **C** | **T** | **C** | **A** | **T** | **C** | **A** | **T** | **C** | **C** | **T** | **C** | **T** | **T** | **C** | **T** | **A** | **G** | **G** |
| **OTS 6** | **C** | **A** | **T** | **C** | **A** | **T** | **C** | **A** | **T** | **C** | **A** | **T** | **C** | **C** | **T** | **C** | **T** | **T** | **C** | **T** | **G** | **A** | **G** |
| **OTS 7** | **C** | **T** | **G** | **C** | **C** | **T** | **C** | **A** | **T** | **C** | **T** | **T** | **C** | **C** | **T** | **C** | **T** | **T** | **C** | **C** | **G** | **A** | **G** |
| **OTS 8** | **C** | **T** | **G** | **C** | **T** | **T** | **T** | **C** | **T** | **C** | **A** | **T** | **C** | **C** | **T** | **C** | **T** | **T** | **C** | **T** | **C** | **A** | **G** |

|  | Site | Potential Off Target Site | Position | No. of Mismatches | Gene | PCR Primer (5’ to 3’) | Product Size(bp) |
| --- | --- | --- | --- | --- | --- | --- | --- |
| Sg2# | OT1 | CTTCCTCATCATCCTCTTCTAAG | chr7:  +142130706 | 1 | TRAK2 | F: TTTGCAACTCACTGCCCTCT  R: CCTTTGTGTCTTTGCCTGGTG | 500 |
|  | OT2 | CTGGCTCATCTTCCTCTTCTTGG | chr3:  -82297932 | 2 | RRS1 | F: ACTGCTACTTCCGCCTCTTTC  R: CGGGACACCAGAGTAAGGAG | 486 |
|  | OT3 | CTGTCTCACCATCCTCTTCTAGG | chrUN0:  -1694385 | 2 | - | F: GACAGAGGGAGGTGAAAGGC  R: GGGTCCTAGCTGTCATGTCG | 605 |
|  | OT4 | CTGCCTCAGTCTCCTCTTCTGGG | chrUN0:  +1712663 | 3 | - | F: CCTTTCGGTTCTCTGTGCTCT  R: CTGTCCATGGCGCTTGTATG | 482 |
|  | OT5 | TTCTCTCATCATCCTCTTCTAGG | chr1:  +72469871 | 3 | - | F: GCACCCTTTCCCCGTAACT  R: GTGGTCCCTGTTGTGGGTTC | 527 |
|  | OT6 | CACCATCATCATCCTCTTCTGAG | chr14:  -157875265 | 3 | - | F: TCGCTTTAGTTTAGGTGGAAGTGT  R: CAGCACTGCAGGTTAGAGGTTTAG | 627 |
|  | OT7 | CTGCCTCATCTTCCTCTTCCGAG | chrUN0:  -549895 | 2 | - | F: TCTTCCTTCCCACAGGCAGT  R: TCTTGCTCTTGCTGGACCTTC | 605 |
|  | OT8 | CTGCTTTCTCATCCTCTTCTCAG | chr17:  -23331789 | 3 | - | F: TTGAGAAACTGCCAAGCTGA  R: GCACAATACTCACCCCTAACC | 613 |

**Table S3. Information regarding the specific primers used for the qPCR.**

| Gene | Primer Sequences (5’ to 3’) | GenBank accession number | Product size (bp) |
| --- | --- | --- | --- |
| MITF | F: CGTGTATGCAGATGGATGATG  R: CAGGTAACGTATTTGCCATTTG | XM_002713310.3 | 118 |
| TYR | F: CATCTTCGATCTGAGTGTCTC  R: TGCCTACAGGGATGACATAG | NM_001082077.1 | 98 |
| TYRP1 | F: CAGAGGATTCTTACAGTCAGGAG  R: CTGGTGGCAATGACAAACTG | NM_001297495.1 | 122 |
| DCT | F: TGTGCATGACCTTCGATAGC  R: CCTGGTGTCAGTTTGTACCTC | NM_001297493.1 | 125 |
